# Role of cleavage at the core-E1 junction of hepatitis C virus polyprotein in viral morphogenesis

**DOI:** 10.1371/journal.pone.0175810

**Published:** 2017-04-24

**Authors:** Véronique Pène, Matthieu Lemasson, Francis Harper, Gérard Pierron, Arielle R. Rosenberg

**Affiliations:** 1 Université Paris Descartes, EA 4474 “Virologie de l’Hépatite C”, Paris, France; 2 CNRS UMR 9196, Institut Gustave Roussy, Villejuif, France; 3 AP-HP, Hôpital Cochin, Service de Virologie, Paris, France; Saint Louis University, UNITED STATES

## Abstract

In hepatitis C virus (HCV) polyprotein sequence, core protein terminates with E1 envelope signal peptide. Cleavage by signal peptidase (SP) separates E1 from the complete form of core protein, anchored in the endoplasmic reticulum (ER) membrane by the signal peptide. Subsequent cleavage of the signal peptide by signal-peptide peptidase (SPP) releases the mature form of core protein, which preferentially relocates to lipid droplets. Both of these cleavages are required for the HCV infectious cycle, supporting the idea that HCV assembly begins at the surface of lipid droplets, yet SPP-catalyzed cleavage is dispensable for initiation of budding in the ER. Here we have addressed at what step(s) of the HCV infectious cycle SP-catalyzed cleavage at the core-E1 junction is required. Taking advantage of the sole system that has allowed visualization of HCV budding events in the ER lumen of mammalian cells, we showed that, unexpectedly, mutations abolishing this cleavage did not prevent but instead tended to promote the initiation of viral budding. Moreover, even though no viral particles were released from Huh-7 cells transfected with a full-length HCV genome bearing these mutations, intracellular viral particles containing core protein protected by a membrane envelope were formed. These were visualized by electron microscopy as capsid-containing particles with a diameter of about 70 nm and 40 nm before and after delipidation, respectively, comparable to intracellular wild-type particle precursors except that they were non-infectious. Thus, our results show that SP-catalyzed cleavage is dispensable for HCV budding *per se*, but is required for the viral particles to acquire their infectivity and secretion. These data support the idea that HCV assembly occurs in concert with budding at the ER membrane. Furthermore, capsid-containing particles did not accumulate in the absence of SP-catalyzed cleavage, suggesting the quality of newly formed viral particles is controlled before secretion.

## Introduction

**Fig 1:**
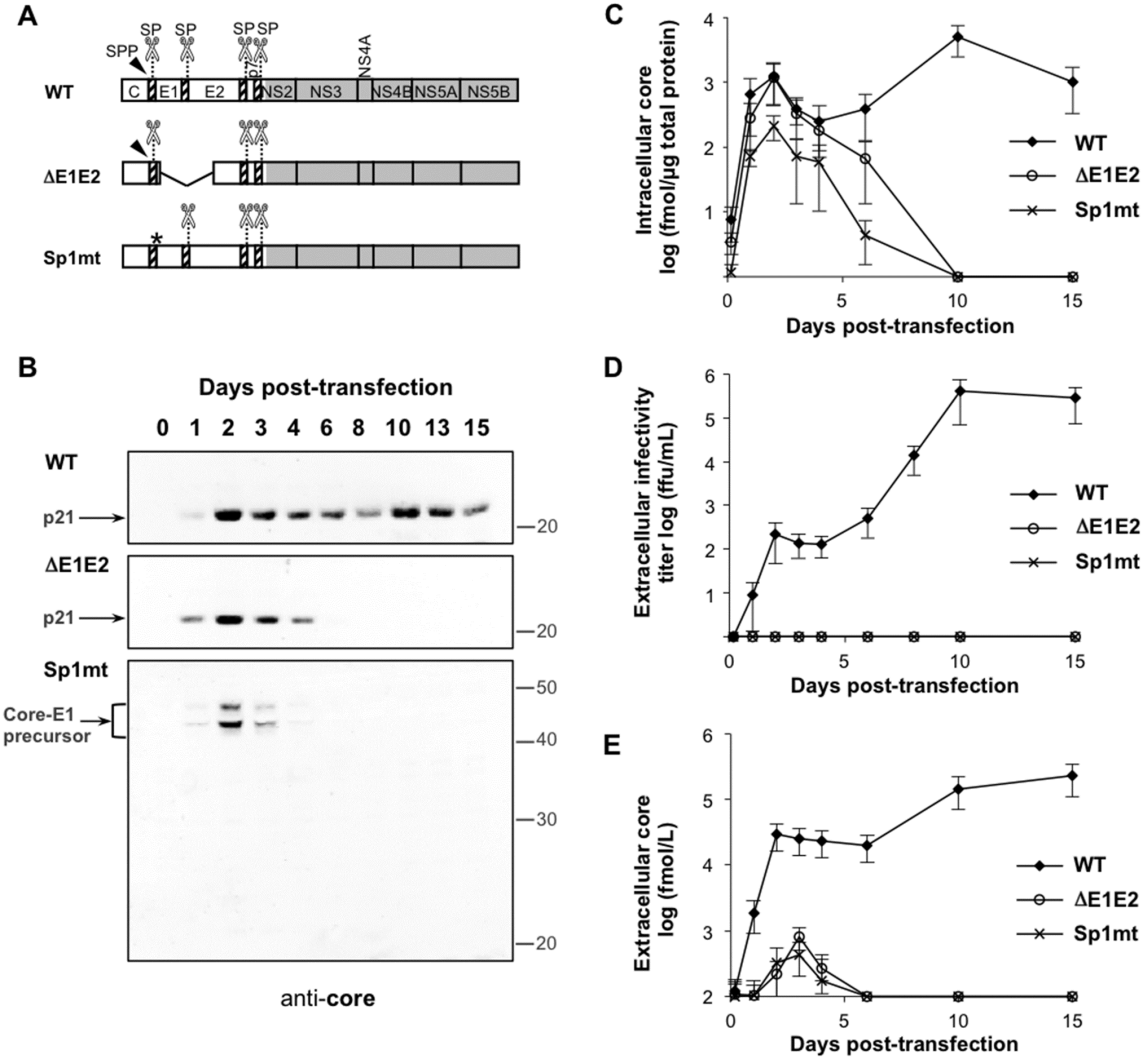
Impact of inhibition of SP-catalyzed cleavage at the core-E1 junction on HCV infectious cycle. Huh-7.5.1 cells were transfected with the full-length HCV RNAs Con1/C3 (WT), Con1/C3/Sp1mt (Sp1mt), or Con1/C3/ΔE1E2 (ΔE1E2). Culture supernatants were harvested and cells were lysed at the indicated days after transfection. (A) Schematic representation of the WT and mutated versions of HCV polyprotein. The scissors and the arrowhead represent the SP and the SPP, respectively. The asterisk indicates the site of Sp1mt mutation. The SPP-catalyzed cleavage cannot occur when SP-catalyzed cleavage at the core-E1 junction is inhibited by Sp1mt mutation. (B) Cell lysates were subjected to western blot analysis with mAb against HCV core protein (C7-50). Positions on blots of protein molecular mass standards are indicated (in kDa). Bands corresponding to the mature core protein (p21) or to different glycoforms of the core-E1 precursor (Core-E1 precursor) are indicated by arrows. A representative western blot is shown. (C) Cell lysates were probed for core antigen using HCV core-specific ELISA. The threshold of detection of this assay is evaluated at 0.3 log fmol per μg of total protein. (D and E) Culture supernatants were probed for infectivity titer (D) and core antigen (E). The thresholds of detection of the infectivity assay and of HCV core-specific ELISA are evaluated at 0.3 log ffu/mL and 2 log fmol/L, respectively. The mean values and standard errors of at least 3 independent experiments are shown.

Hepatitis C virus (HCV), a major causative agent of chronic hepatitis in human, was the first described member of the genus *Hepacivirus* belonging to the *Flaviviridae* family. HCV is an enveloped virus with a single-strand positive RNA genome. This genome encodes a single polyprotein precursor that undergoes a series of proteolytic cleavages to generate functional viral proteins (Fig 1A). HCV structural proteins, which include core protein and envelope glycoproteins E1 and E2, derive from the N-terminal portion of the polyprotein *via* cleavages catalyzed by proteases of the host cell endoplasmic reticulum (ER). HCV core protein is the most N-terminal component of the viral polyprotein, and terminates with E1 signal peptide [1]. This peptide directs the nascent polypeptide chain to the ER membrane, and induces translocation of the downstream E1 region into the ER lumen, while leaving the core protein region on the cytosolic side. Cleavage by host cell signal peptidase (SP) at the luminal side of the ER separates E1 from p23, the so-called immature form of core protein containing 191 residues [2, 3]. This complete form of HCV core protein is anchored in the ER lipid bilayer by the C-terminal signal peptide [4]. Subsequent intramembrane cleavage catalyzed by signal-peptide peptidase (SPP) generates p21, the so-called mature form of core protein, which is devoid of signal peptide and is free for trafficking to lipid droplets (LDs). Importantly, it is now established that SP-catalyzed cleavage at core-E1 junction is a prerequisite for SPP-catalyzed cleavage [5, 6].

HCV structural proteins form the viral particle, whose morphogenesis is schematically divided into several steps: initiation of assembly, budding, maturation, and secretion leading to egress. However, HCV morphogenesis is not fully understood, in particular considering the timing and localization of each step. The initiation of HCV morphogenesis should be a tightly synchronized event in order to switch from replication to assembly and bring together the virus structural proteins and the viral genome synthesized within the replication complex [7–9]. HCV core protein has been attributed a critical role in this process, as the mature form of the protein preferentially relocates to the surface of LDs and, once there, recruits the virus non-structural (NS) proteins and replication complex [10, 11]. The localization of core protein at the LD surface was reported to be necessary for the production of infectious HCV, and LDs have been proposed to act as platforms for the initiation of viral assembly [11–13], but no unified picture of the assembly process has been established. By homology with other members of the *Flaviviridae* family, HCV budding is proposed to occur towards the ER lumen by envelopment of the nucleocapsid through the ER membrane containing E1 and E2 envelope glycoproteins [14]. A current, most widely accepted, model for HCV morphogenesis involves the formation of a nucleocapsid at the LD surface that would subsequently be enveloped at the ER membrane [15]. However, it remains unclear whether initiation of assembly occurs on the LD side, ER side, or at the LD/ER interface, and whether assembly and budding are sequential or simultaneous steps [14, 16]. The driving force of HCV budding also remains largely elusive, depending on HCV envelope glycoproteins, or core protein, or both, possibly in conjunction with host cell factors [17]. Once the budding process completed, viral particles would undergo a quality control and be either degraded or matured and transported within the cell leading to egress in the extracellular medium [17–20]. A peculiar feature of HCV infectious particles is their association with components of very-low-density lipoproteins (VLDLs) in hybrid structures referred to as lipo-viro-particles (LVPs) [21, 22], and hence another aspect to be taken into consideration when elaborating a model for HCV morphogenesis is the possible requirement of the VLDL biogenesis pathway [18, 23–25].

After the cloning of HCV genome in 1989 [26], it took almost two decades before a culture system has been made available for this virus, using Huh-7 cell sublines and JFH1, a full-length HCV genome with exceptional replication capabilities in vitro [27]. The importance for HCV infection cycle of each of the two sequential cleavages that lead to generation of mature core protein has been established in this system [5, 28–32]. Indeed, inhibition of SPP-catalyzed cleavage of HCV core protein by various approaches significantly decreased production of infectious HCV particles [28–32]. We also previously showed that mutations in HCV genome inhibiting SP-catalyzed cleavage at the core-E1 junction completely abolish the production of infectious particles [5]. While the HCV culture system has been extensively used to identify determinants of viral morphogenesis [33], only two publications reported the visualization of putative viral particles in situ within the cells [11, 34]. Moreover, there are still no pictures available of assembly and/or budding in Huh-7 infected cells, pointing to the rarity and/or rapidity of such events. In this context, heterologous expression systems have been used to study the first steps of HCV morphogenesis, and transfection of BHK-21 cells with a Semliki forest virus (SFV)-based replicon encoding HCV structural proteins remains the only model allowing the visualization of budding events in the ER of mammalian cells by electron microscopy (EM) [35]. Using this system, we previously showed that inhibition of SPP-catalyzed cleavage of core protein not only did not prevent initiation of HCV budding, but instead tended to promote the budding process [36].

Here we have investigated at what step(s) of HCV infectious cycle SP-catalyzed cleavage at the core-E1 junction is required. For this purpose, we inhibited this cleavage by introducing mutations at the core-E1 junction of an HCV sequence of genotype 1b, the most frequent genotype in Western countries, and analyzed the impact of these mutations (i) on the initiation of budding in BHK-21 cell system and (ii) on the infectious cycle in Huh-7.5.1 cell system.

## Material and methods

### Plasmid constructs and site-directed mutagenesis

The plasmid pFK-Con1/C3 [37] was a kind gift from R. Bartenschlager (University of Heidelberg, Germany) (Fig 1A). Oligonucleotide-directed mutagenesis was performed as described previously to produce plasmids pFK-Con1/C3/Sp1mt and pFK-Con1/C3/ΔE1E2 [5]. To generate pFK-Con1/C3/ΔE1E2, the HCV sequence coding for amino acids 216 to 567 was deleted by use of the following oligonucleotide primer: 5’-GCAAGCATTGTGTATGAGCCGTGTAACATCGGGGGGATCGGC-3’. The plasmids pSFV-*lac*Z, pSFV-*HCV*1b, pSFV-*HCV*1b/Sp1mt and pSFV-*HCV*1b/Sp2mt have been described elsewhere [5].

### Cell culture-based expression systems

Expression of full-length HCV replicons in human hepatoma cells was achieved as previously described [5]. Briefly, the different versions of pFK-Con1/C3 were linearized at the *Pvu*I site, and served as templates for *in vitro* transcription. Huh-7.5.1 cells (a kind gift from F.V. Chisari, Scripps Research Institute, La Jolla, CA, 2005) [38] were electroporated with *in vitro* transcribed RNAs, plated at a density of 5 x 10^4^ cells per cm^2^, and cultured for the indicated days. Cells were lysed adequately for western blotting experiments, for quantification of HCV replication, for quantification of core antigen, for extraction of intracellular particles or for the membrane protection assay (detailed below and in S1 Appendix). Culture supernatants were passed through a 0.2-μm filter and further analyzed for cytotoxicity, HCV RNA, core antigen, or infectivity titer.

Expression in BHK-21 mammalian cells (a gift of M. Bouloy, Pasteur Institute, Paris, France, 2004) was achieved as previously described [36]. Briefly, the different versions of pSFV-*HCV*1b plasmids or pSFV-*lac*Z were linearized at the *Spe*I site, and used as templates for *in vitro* transcription. BHK-21 cells were electroporated with the resulting RNAs and immediately plated and cultured for 20 h in growth medium before analysis by western blotting, or by immunofluorescence or electron microscopy.

### Western blotting

Western blotting was performed mainly as described previously [36]. Briefly, cell lysates in reducing sample buffer were electrophoresed in SDS-12% polyacrylamide gels under reducing conditions, in parallel with protein molecular mass standards (MagicMark^™^, Invitrogen). Subsequently, proteins were transferred onto nitrocellulose membranes (Amersham Protran, GE Healthcare). Membranes were blocked with PBS containing 0.05% Tween 20 and 2% ECL Prime Blocking Agent (GE Healthcare), and probed 1h with primary antibodies directed against HCV core protein (C7-50, Alexis Biochemicals), HCV E2 glycoprotein (AP33, kindly provided by A. H. Patel, MRC Virology Unit, Institute of Virology, Glasgow, UK), HCV NS3 protein (8 G-2, Abcam), or calnexin (C4731, Sigma-Aldrich). Excess antibody was removed with five washes, and the second-step antibody (DyLight 800 labeled goat anti-mouse or DyLight 680 labeled goat anti-rabbit, KPL) diluted 1:15,000 in blocking buffer was allowed to bind for 1 h. After several washes, protein bands were visualized using LI-COR-Odyssey infra-red scanner (LI-COR).

### Quantification of core antigen and titration of infectivity

HCV core antigen levels were quantified by enzyme immunoassay using the Ortho^R^ HCV Ag ELISA kit (Ortho-Clinical Diagnostics) in accordance with the manufacturer’s instructions. For quantification of intracellular core antigen levels, cells were lysed for 10 min at 4°C in phosphate buffered saline (PBS) containing 1% Triton X-100 and 0.2 mg/mL protease inhibitor 4-(2-aminoethyl)benzesulfonyl fluoride (ICN Biochemicals) and cell debris were pelleted by centrifugation for 10 min at 20 000 x g at 4°C and discarded. Intracellular core antigen levels were normalized to total intracellular protein amount, determined by Bradford assay (Bio-Rad) as specified by manufacturer’s instructions.

Infectivity titers were determined by focus-formation assay as previously described [5], and expressed as focus-forming units (ffu) per mL or ffu per μg of total proteins in cell-culture supernatants and cell lysates, respectively.

### Electron microscopy (EM) analysis of BHK-21 cells

For ultrastructural studies and immuno-EM experiments, BHK-21 cells transfected with SFV-based replicon RNAs were treated as previously described [39]. Briefly, for in situ visualization of HCV-like particle budding, cells were fixed with 1.6% glutaraldehyde, post-fixed with 1% osmium tetroxide, washed, and stained with 2% uranyl acetate. Cells were then dehydrated and embedded in Epon 812. For observation, ultra-thin sections were contrasted with 4% uranyl acetate and lead citrate and examined under a Zeiss 902 electron microscope at 80 kV. Areas of convoluted ER with HCV-like particles budding toward the dilated lumen were measured using the NIH ImageJ version 1.63 software, and the number of budding particles arrested at early or late stages of the budding process was counted. For immuno-EM experiments, cells were fixed with 4% formaldehyde, dehydrated, and embedded in Lowicryl K4M at low temperature under long-wavelength UV light. Ultrathin sections of Lowicryl-embedded material were used for labeling with monoclonal antibodies (mAb) directed against HCV core protein (1856, Virostat) or against HCV E2 glycoprotein (AP33) [40], followed by incubation with a goat anti-mouse or anti-rabbit IgG conjugated to gold particles, 10 nm in diameter. Sections were contrasted with uranyl acetate.

### Extraction of intracellular particles

Two days post-transfection, Huh-7.5.1 cells from six 100-mm dishes were washed three times in PBS and pelleted by centrifugation at 100 x g for 5 min. Pelleted cells were lysed by four freeze-thaw cycles, using dry ice plus ethanol for freezing and a 37°C water bath for thawing. Pellet was resuspended in 0.6 mL TNE buffer (50 mM Tris-HCl [pH 7.4], 100 mM NaCl, 0.5 mM EDTA). After centrifugation for 5 min at 16,000 x g to pellet cell debris, supernatant was collected and further analyzed for infectivity titer or subjected to isopycnic ultracentrifugation.

### Membrane protection assay

Two days post-transfection, Huh-7.5.1 cells were washed three times and scrapped in PBS, then pelleted by centrifugation at 1000 x g for 1 min. Pelleted cells were resuspended in proteinase K buffer (50 mM Tris-HCl [pH 8.0], 10 mM CaCl_2_, 1 mM DTT), lysed by five freeze-thaw cycles, using dry ice plus ethanol for freezing and a 37°C water bath for thawing. The resulting lysate was separated into three samples: one sample was left untreated, the second sample was treated with 5 μg/mL proteinase K (Merck), and the third sample was subjected to the same proteinase K treatment in the presence of 1% Triton X-100. After 1 h on ice at 4°C, the digestion was terminated by addition of 5 mM 4-(2-aminoethyl)benzesulfonyl fluoride and a further 10-min incubation of the samples on ice. Reducing sample buffer 4X was then added and the samples were heated at 95°C for 10 min before proceeding to western blot analysis with mAb against HCV core protein (C7-50) as described above, or dot blotting for quantification. For dot blotting, the samples were loaded directly onto nitrocellulose membranes. Membranes were allowed to dry, subsequently washed several times in water and PBS, and immunoblotted with mAb against HCV core protein (C7-50) as described for western blot analysis. Quantification of signal intensities was performed using ImageJ software.

### EM analyses of viral particles recovered from cell lysates

To partially purify viral particles from cell lysates, isopycnic ultracentrifugation was performed essentially as previously described [41] with minor modifications, as follows. A 9-mL continuous iodixanol gradient was prepared with Gradient Former 485 (Bio-Rad) by using 10% and 40% (wt/vol) OptiPrep (Axis-Shield) solutions in gradient buffer (10 mmol/L HEPES [pH 7.55], 0.02% bovine serum albumin [BSA]). To maintain iso-osmotic conditions throughout the gradient, a reverse gradient of sodium chloride was made by adding 120 and 50 mmol/L NaCl to the 10% and 40% OptiPrep solutions, respectively. This continuous iodixanol gradient was overlaid with a 1-mL layer of 8% (wt/vol) OptiPrep, which was covered with a 0.6-mL layer of 6% (wt/vol) OptiPrep in gradient buffer. The cell lysate sample (typically 0.5 mL supplemented with 0.5 mL of culture medium) was loaded onto this preformed gradient, and isopycnic ultracentrifugation was performed in a Beckman SW41Ti rotor (Beckman Coulter) at 110,000 x g for 20 hours at 4°C before fractionation from the top of the tube into 8 fractions of 1.4 mL. The density of each fraction was determined by measuring the refractive index of a 10-μL aliquot with an Abbe refractometer (Atago) at a constant temperature of 20°C.

For visualization of intracellular particles, fractions with densities ranging from 1.12 to 1.13 g/mL were pooled and fixed with either 2% glutaraldehyde or 0.4–1.3% formaldehyde in H_2_O for direct examination and immuno-labeling experiments, respectively. For direct examination, preparations were loaded onto carbon-coated grids for 2 min. For immunogold labeling, a droplet of 4% formaldehyde was loaded on the grids for 5 min for completion of fixation. Grids were blocked with PBS containing 5% BSA for 2 min and probed for 20 min with mAb against HCV core protein (C7-50), diluted at 1:20 in PBS containing 0.1% Triton X-100 and 1% BSA. Excess mAb was removed with three washes in PBS, and grids were incubated 20 min with the secondary antibody (10 nm-gold-particle-conjugated mouse-specific antibody) diluted 1:50 in PBS containing 0.1% Triton X-100 and 1% BSA. For both direct examination and immuno-labeling experiments, negative staining was performed by adding 3 successive droplets of 1% uranyl acetate before grids were examined under a Zeiss 902 electron microscope at 80 kV.

## Results

### SP-catalyzed cleavage at core-E1 junction is necessary for the HCV infectious cycle

To examine the role of SP-catalyzed cleavage at the core-E1 junction in the HCV infectious cycle, we used the full-length HCV replicon Con1/C3, a JFH1-derived chimera encoding HCV structural proteins of genotype 1b which allows the production of infectious viral particles in the human hepatic cell line Huh-7 [37], and the combination of mutations Sp1mt, previously shown to abolish cleavage at the core-E1 junction in this HCV sequence [5] (Fig 1A). As a negative control, we deleted a large in-frame portion within the E1E2 coding sequence to generate the Con1/C3/ΔE1E2 construct, expected to allow replication of the viral genome while preventing assembly of viral particles, as is the case with JFH1/ΔE1E2 replicon [27].

Huh-7.5.1 cells were transfected with the wild-type (WT) or mutated versions of HCV RNAs Con1/C3, and lysed at various time points post-transfection for western blot analysis with mAb against HCV core protein (Fig 1B). In cells transfected with WT or ΔE1E2 constructs, core protein was detected as a unique band with an apparent molecular mass of 21 kDa, corresponding to the mature protein usually referred to as p21. Thus, p21 is the predominant form of core protein at steady state in cells expressing HCV replicons bearing a WT sequence at the core-E1 junction. By contrast, in cells transfected with the Sp1mt construct, mAb against HCV core protein revealed different glycosylation forms of the core-E1 precursor which we described previously [5]. Nevertheless, reprobing the blots with mAb against HCV E2 envelope glycoprotein confirmed that this glycoprotein was correctly processed from the Sp1mt-mutated polyprotein (Figure A, upper panel, in S1 Fig). Thus, in this system the combination of mutations Sp1mt efficiently inhibits SP-catalyzed cleavage at the core-E1 junction specifically.

To evaluate core protein expression levels, we used a commercial ELISA for HCV core antigen (Fig 1C). At 4 h post-transfection the intracellular amounts of core antigen were similar in lysates of cells transfected with HCV RNAs Con1/C3 or Con1/C3/ΔE1E2, indicating comparable transfection efficiency, but were slightly lower in cells transfected with Con1/C3/Sp1mt. Although this was consistent with our previous findings that the mutated core-E1 precursor is not as stable as the WT core protein [5], it was important to verify the levels of HCV replication and translation achieved upon transfection with Con1/C3/Sp1mt compared to the control constructs. At day 1 post-transfection the amounts of both negative-strand HCV RNA, considered a hallmark of HCV genome replication (Figure B in S1 Fig), and HCV non-structural protein NS3, used as a marker of HCV polyprotein translation and processing (Figure A in S1 Fig), were indeed similar for all three constructs, which suggested comparable transfection efficiency and further showed that the mutations do not significantly affect replication or translation of the viral genome. However, in cells transfected with HCV RNAs Con1/C3/ΔE1E2 or Con1/C3/Sp1mt, the amounts of negative-strand HCV RNA (Figure B in S1 Fig), NS3 protein (Figure A in S1 Fig), and core protein (Fig 1B) or antigen (Fig 1C) rapidly decreased after 4 days post-transfection, the latter marker becoming undetectable at day 10 post-transfection. In the case of WT replicon, by contrast, all markers were readily detected throughout the 15-day kinetics (Fig 1B and 1C, and Figures A and B in S1 Fig), and in particular, intracellular amounts of HCV core antigen slightly diminished at 4 days post-transfection and then increased again with a peak on day 10 post-transfection at the approximate mean value of 5 x 10^3^ fmol of core antigen per μg of total protein (Fig 1C). These results suggest that only the WT replicon is able to establish a chronic infection and that SP-catalyzed cleavage at core-E1 junction is required for the completion of HCV infectious cycle at a step distinct from translation and replication of the viral genome.

### SP-catalyzed cleavage at core-E1 junction is a prerequisite for HCV particle release

To directly address the hypothesis that SP-catalyzed cleavage at core-E1 junction is required for infectious virus production, we measured the infectivity titer in culture supernatants of cells transfected with HCV RNAs Con1/C3, Con1/C3/ΔE1E2, or Con1/C3/Sp1mt (Fig 1D). In the case of WT, as expected, infectious viral particles were detected as early as day 1 post-transfection, with the infectivity titer reaching a peak at the mean value of 4 x 10^5^ ffu/mL on day 10. By contrast, infectivity was undetectable at any time point of the 15-day kinetics in supernatants from cells transfected with HCV RNAs Con1/C3/ΔE1E2 or Con1/C3/Sp1mt, indicating that no infectious viral particles were released.

The lack of extracellular infectivity observed upon inhibition of SP-catalyzed cleavage might result from a defect in the release of viral particles from the producing cell, or from a defect in the infectivity of released viral particles such as inefficient entry or uncoating in the target cell. To distinguish between these two hypotheses, we investigated whether physical viral particles were released from transfected cells irrespective of their infectivity. Measurement of HCV core antigen levels in cell-culture supernatants was used as a marker of physical viral particle release (Fig 1E). Extracellular core antigen was detected as early as 1 day after transfection with WT replicon, and reached a peak of 1.5 x 10^5^ fmol/L on day 10 post-transfection. By contrast, in case of transfection with the Sp1mt-mutated replicon, core antigen was below the threshold of detection of the assay (2 log fmol/L) for most of the time course except for days 2 to 4, when some core antigen that was not associated with infectivity was detected at levels even lower than those measured in case of the assembly-defective mutant Con1/C3/ΔE1E2 (compare Fig 1E and 1D). This small quantity of core antigen probably resulted from cell lysis because transfection was followed by a phase of cell mortality during the first few days, as shown by lactate-dehydrogenase leakage at levels comparable for all constructs (Figure C in S1 Fig) [27]. We conclude that SP-catalyzed cleavage at core-E1 junction is necessary for release of infectious or non-infectious HCV particles.

### Inhibition of SP-catalyzed cleavage at core-E1 junction promotes initiation of HCV budding

That no viral particles were released from Huh-7.5.1 cells transfected with full-length Con1/C3/Sp1mt replicon suggested that HCV budding might be compromised when SP-catalyzed cleavage is not allowed to occur. Unfortunately, the full-length HCV replicon/Huh-7 cell system does not allow the visualization of budding events. Therefore, we took advantage of the SFV-based replicon/BHK-21 cell system as a budding assay. Indeed, upon expression of a partial HCV polyprotein encompassing all three structural proteins, HCV-like particle budding events can be visualized at steady state in the ER lumen of these mammalian cells [35]. In a previous publication, we showed that either Sp1mt or another combination of mutations, Sp2mt, efficiently inhibit the SP-catalyzed cleavage at core-E1 junction in this system, as BHK-21 cells transfected with the corresponding constructs expressed different glycosylation forms of the core-E1 precursor and aberrant degradation products but not the processed core and E1 proteins [5]. Here we further verified that E2 was correctly processed from these mutated polyproteins, the different glycosylation forms of this envelope glycoprotein being expressed at comparable levels in BHK-21 cells transfected with the WT or mutated versions of recombinant SFV-*HCV*1b RNAs (S2 Fig). As expected, immunofluorescence experiments using mAb against HCV core protein showed a reticular pattern colocalizing with calnexin staining in BHK-21 cells transfected with recombinant SFV-*HCV*1b/Sp1mt or SFV-*HCV*1b/Sp2mt RNAs, confirming the retention of core protein precursors at the ER membrane, whereas in cells transfected with their WT counterpart core protein was localized mainly at the surface of LDs, which also appeared tightly aggregated in the perinuclear region (S3 Fig).

**Fig 2:**
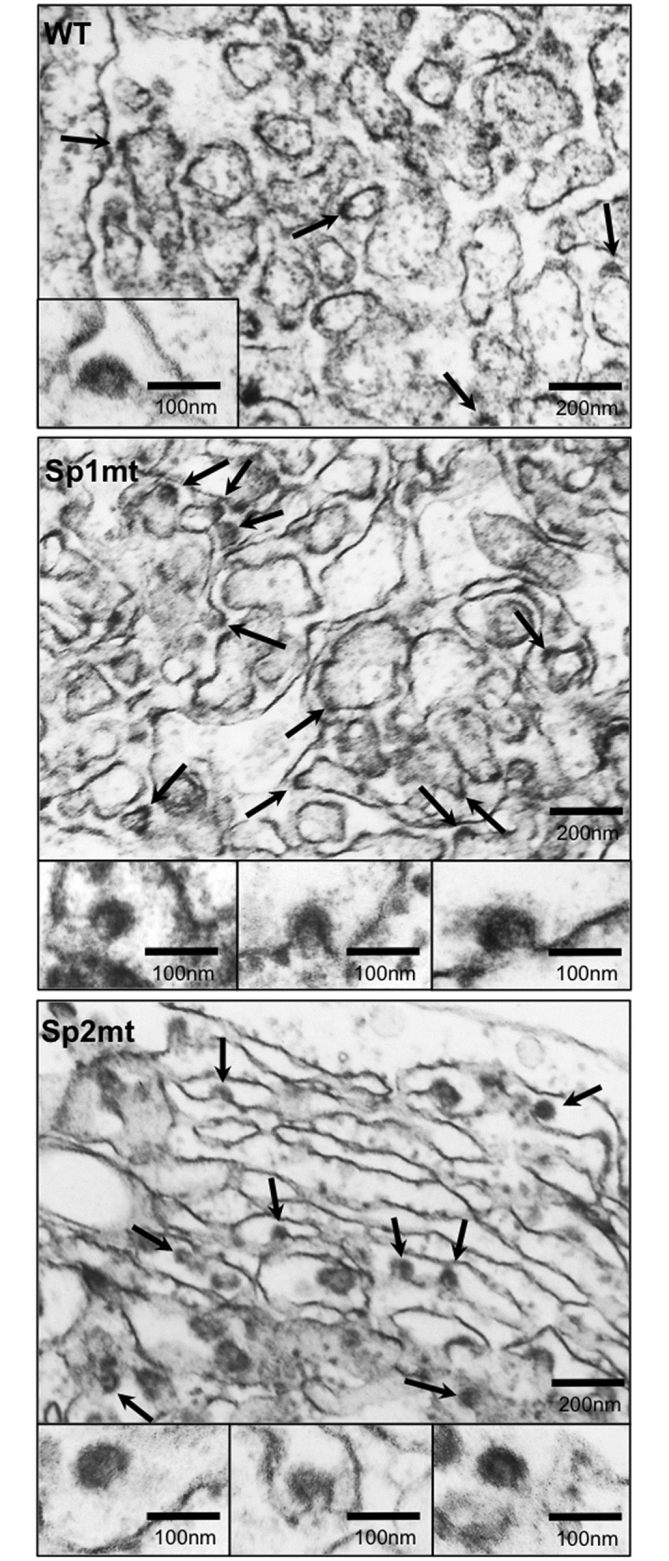
In situ EM visualization of HCV-like particle budding. Representative electron micrographs of ultra-thin sections of BHK-21 cells electroporated with the recombinant RNAs SFV-*HCV*1b (WT), SFV-*HCV*1b/Sp1mt (Sp1mt), or SFV-*HCV*1b/Sp2mt (Sp2mt). Some budding events are indicated on electron micrographs at low magnification (arrows). High-magnification images of such budding events are shown in insets.

**Fig 3:**
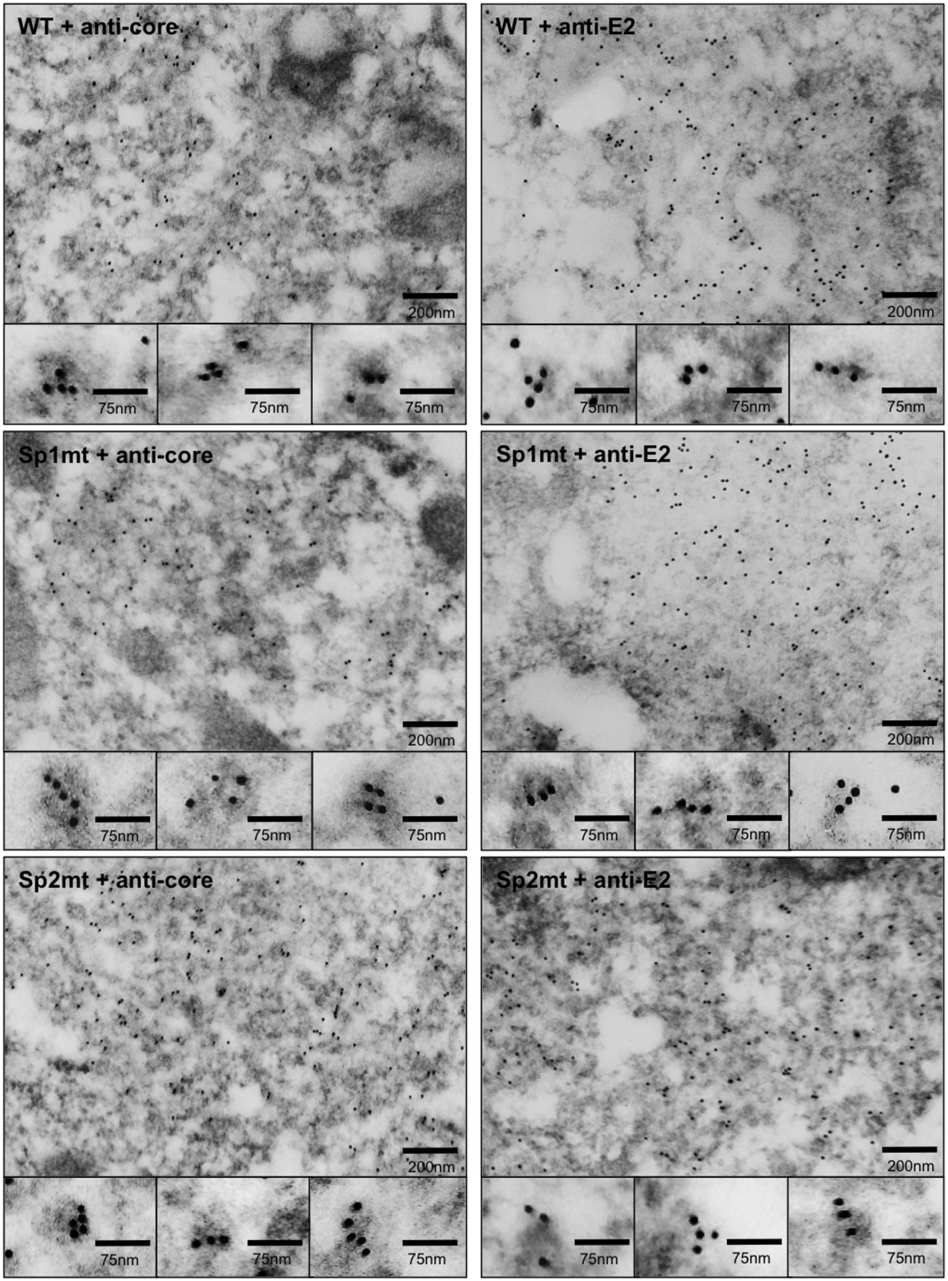
In situ EM detection of HCV-like particle budding after immunogold labeling. Shown are representative electron micrographs of ultra-thin sections of BHK-21 cells electroporated with the recombinant RNAs SFV-*HCV*1b (WT), SFV-*HCV*1b/Sp1mt (Sp1mt), or SFV-*HCV*1b/Sp2mt (Sp2mt), after immunogold labeling with mAbs against HCV core protein (+ anti-core) or HCV E2 envelope glycoprotein (+ anti-E2). High-magnification images of gold-labeled budding events are shown in insets.

To determine whether the mutations that prevented SP-catalyzed cleavage at core-E1 junction also prevented the initiation of viral budding, we analyzed BHK-21 cells transfected with the WT or mutated versions of recombinant SFV-*HCV*1b RNAs by EM (Fig 2; see also Figure A in S4 Fig, for control cells transfected with the irrelevant SFV-*lac*Z RNA) as described previously [36]. Cells transfected with WT construct showed convoluted membranes with dilated lumen derived from the ER, and assembly of HCV structural proteins at the ER membrane was visualized as electron-dense patches, which appeared in some cases as hemispherical structures, 60 nm in diameter, budding towards the dilated lumen, as previously described [35, 36]. Surprisingly, EM examination of cells transfected with recombinant SFV-*HCV*1b/Sp1mt or SFV-*HCV*1b/Sp2mt RNAs revealed the presence of areas of convoluted ER with a large number of HCV-like particles budding towards the dilated lumen (Fig 2). Most importantly, core and E2 proteins were detected in areas of convoluted ER by immunogold labeling using specific mAbs, some viral buds appearing nicely and specifically decorated with gold particles (Fig 3), whereas no specific gold labeling was seen in cells transfected with the control SFV-*lac*Z RNA (Figure B in S4 Fig). Various types of buds were observed (Figs 2 and 3, see the high-magnification images), and classified as either “early buds” (hemispherical structures with no neck) or “late buds” (particles that seemed to have moved forwards up to a late stage of budding and were only tethered to the ER membrane by a stalk) in accordance with the criteria used in a previous publication [36]. Interestingly, areas of convoluted ER displayed a greater density of budding particles in cells transfected with either of the mutated constructs as compared to WT, and the majority of the viral buds also appeared as “late buds” (Table 1). We conclude that inhibition of SP-catalyzed cleavage at the core-E1 junction not only did not prevent the initiation of budding, but instead tended to facilitate the budding process.

**Table 1:** Estimation of budding efficiency.

	Surface analyzed (μm^2^)^a^	Density of budding particles per μm^2^		
	Total	Early buds	Late buds	Total
WT	41.13	2.20	0.09	2.29
Sp1mt	20.38	4.98	15.08	20.06
Sp2mt	24.80	4.78	10.59	15.37

^*a*^ Areas of convoluted ER with HCV-like particles budding towards the dilated lumen were analyzed in at least five different cells.

### SP-catalyzed cleavage at core-E1 junction is dispensable for the formation of intracellular capsid-containing particles

**Fig 4:**
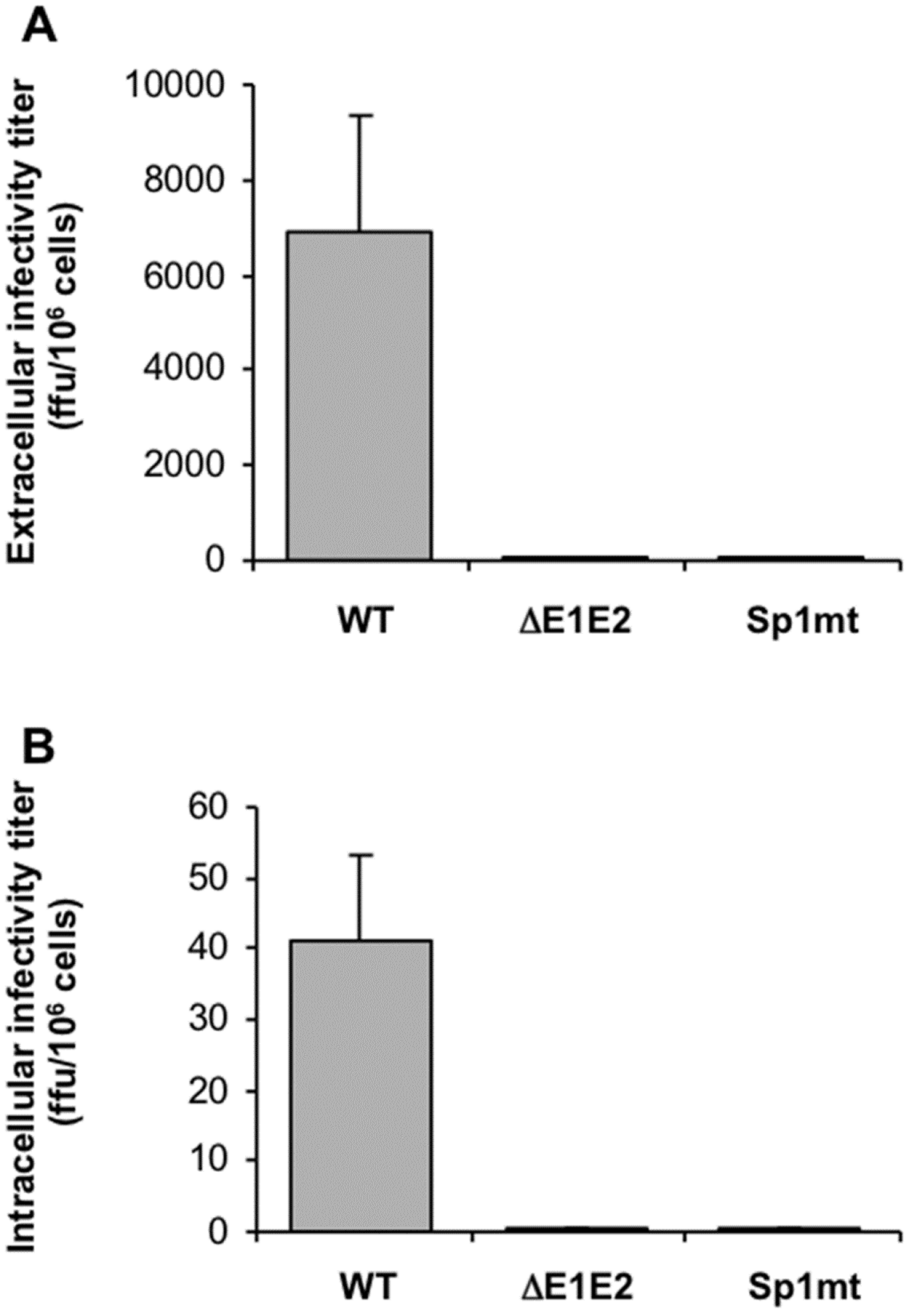
Titration of extracellular and intracellular infectivity. Huh-7.5.1 cells were transfected with the full-length HCV RNAs Con1/C3 (WT), Con1/C3/ΔE1E2 (ΔE1E2), or Con1/C3/Sp1mt (Sp1mt). Two days post-transfection, (A) culture supernatants were harvested and probed for extracellular infectivity titers, and (B) cells were lysed by freeze-thaw cycles and probed for intracellular infectivity titers. Mean values of four independent experiments are shown and the standard errors of the means are presented. The threshold of detection of the intracellular infectivity assay is evaluated at 2 ffu per 10^6^ transfected cells.

SP-catalyzed cleavage at core-E1 junction is a prerequisite for the release of infectious and non-infectious particles, yet is dispensable for initiation of budding. These results suggested the hypothesis that intracellular formation of viral particles that would not be released might occur even when this cleavage is not allowed to occur. However, because HCV budding is abortive in the SFV-based replicon/BHK-21 cell system [35], the completion of this process cannot be studied by this experimental approach. We thus used the full-length HCV replicon/Huh-7 cell system to elucidate whether SP-catalyzed cleavage at core-E1 junction is necessary for intracellular formation of infectious virus. As shown in Fig 1, intracellular levels of core antigen reached a maximum 2 days after transfection of Huh-7.5.1 cells with the full-length Con1/C3/Sp1mt replicon. We thus chose this time point to assess infectivity in cell lysates in parallel with the corresponding cell-culture supernatants (Fig 4). As expected, infectivity was found associated with cells transfected with the WT Con1/C3 replicon, corresponding to 0.6% of the infectivity found in the cell-culture supernatant. By contrast, lysates of cells transfected with either Sp1mt- or ΔE1E2-mutated replicon were not infectious. Thus, no infectious particles were formed upon inhibition of SP-catalyzed cleavage at core-E1 junction, but non-infectious particles might still be formed within the cells.

**Fig 5:**
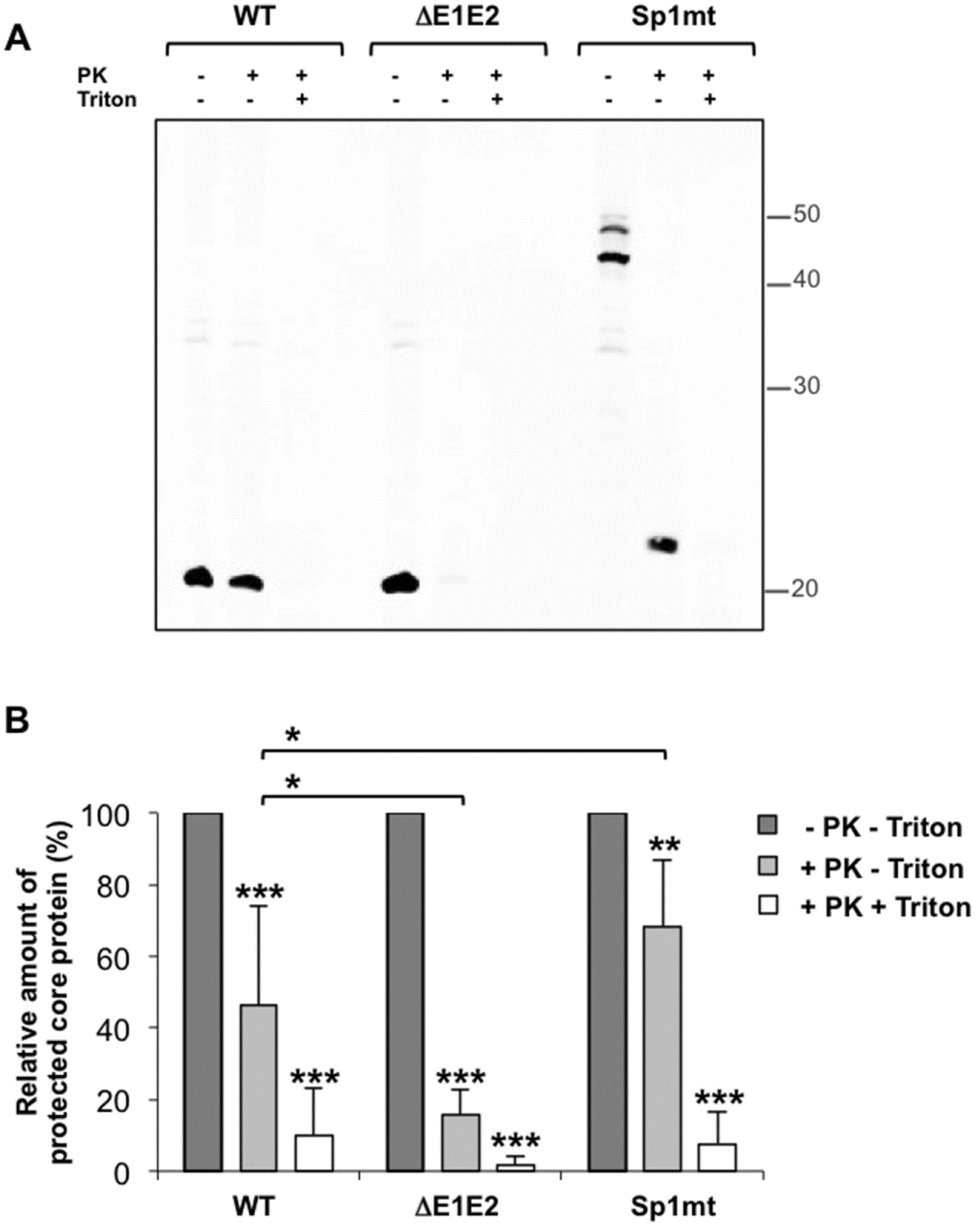
Core protein envelopment analysis by membrane protection assay. Huh-7.5.1 cells were transfected with the full-length HCV RNAs Con1/C3 (WT), Con1/C3/ΔE1E2 (ΔE1E2) or Con1/C3/Sp1mt (Sp1mt). Two days post-transfection, cells were lysed by freeze-thaw cycles and left untreated or treated with 5 μg/mL proteinase K (PK) in the presence or absence of 1% Triton X-100 (Triton). The samples were subjected to (A) western blot analysis with mAb against HCV core protein (a representative western blot is shown) or (B) dot blot for quantification of signal intensities with ImageJ (values were normalized to untreated samples). The mean values and standard errors of four independent experiments are shown. *, P comprised between 0.05 and 0.01; **, P comprised between 0.01 and 0.001; ***, P below 0.001.

If enveloped particles were formed, then their core protein content should be protected from proteolytic digestion unless a detergent was added to dissolve the membrane envelope. We used a membrane protection assay based on this principle to assess the degree of envelopment of core protein (Fig 5). In cells transfected with the WT Con1/C3 replicon, a mean 46% of core protein was resistant to digestion by proteinase K and this value decreased to 10% when membranes were concomitantly dissolved with Triton X-100. As expected, the percentage of proteolysis-resistant core protein was significantly lower in cells transfected with the control Con1/C3/ΔE1E2 replicon, and comparable with the data reported with another JFH1-derived chimeric virus [42]. By contrast, in cells transfected with Sp1mt-mutated replicon the percentage of core protein protected from proteolysis reached 68.4 ± 18.7% in the absence of Triton X-100, indicating that the degree of core protein envelopment was even significantly higher than in the case of WT (Fig 5B). Most importantly, the form of core protein detected after proteolysis was a unique band with an apparent molecular weight of 23 kDa, suggesting that only the core moiety of the mutated core-E1 precursor was protected from proteolysis whereas the E1 moiety was exposed to proteinase K, consistent with the expected topology of core-E1 precursor incorporated in an enveloped viral particle (Fig 5A). These results suggested that core protein envelopment into intracellular viral particles occurred within the cell even when SP-catalyzed cleavage at core-E1 junction was not allowed to occur.

**Fig 6:**
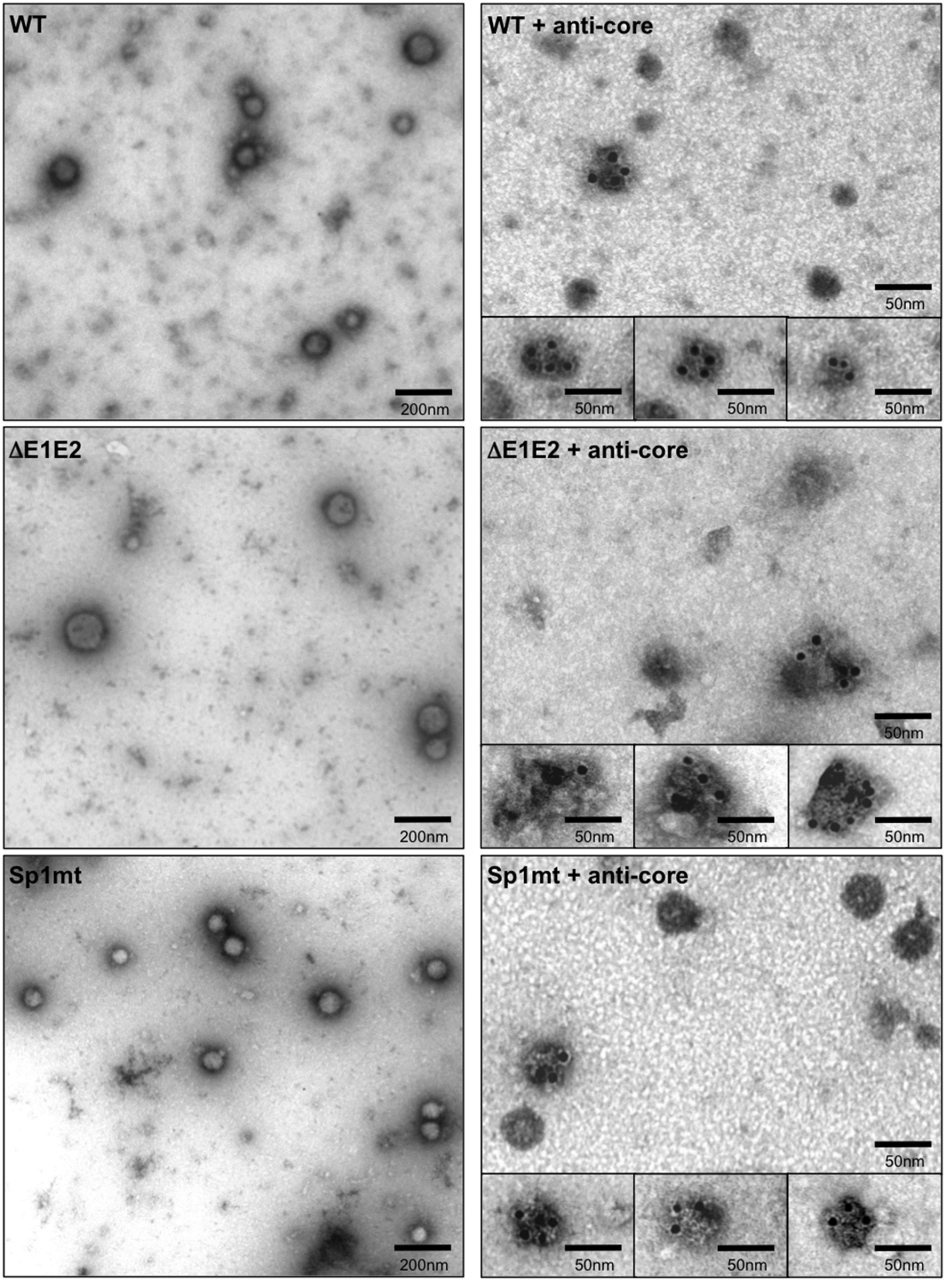
EM visualization of intracellular HCV particles. Huh-7.5.1 cells were transfected with the full-length HCV RNAs Con1/C3 (WT) or Con1/C3/ΔE1E2 (ΔE1E2) or Con1/C3/Sp1mt (Sp1mt). Two days post-transfection, cells were lysed by freeze-thaw cycles and cell lysates were loaded onto iodixanol gradients. After isopycnic ultracentrifugation, fractions with densities ranging from 1.12 to 1.13 g/mL were pooled, and directly subjected to EM visualization after negative staining (left panels) or delipidated with 0.1% Triton X-100 and subjected to immunogold labeling with mAb against HCV core protein before negative staining and EM visualization (right panels).

We felt that it was important to visualize these viral particles and provide evidence of their capsid content. In an attempt to partially purify intracellular HCV particles, transfected cell lysates were loaded onto an iodixanol gradient and fractions were collected after isopycnic ultracentrifugation. Intracellular infectious HCV particles of JFH1 or another JFH1-derived chimeric virus were previously shown to peak at densities close to 1.13 g/mL, representing intracellular precursors of infectious HCV particles secreted into the culture supernatant [18, 25, 43], therefore we analyzed fractions with a density of 1.13 g/mL by EM after negative staining (Fig 6). In the case of WT, this analysis showed the presence of particles with a diameter ranging from 60 to 80 nm (Fig 6, WT, left panel). After delipidation, smaller particles resembling capsids were detected with a diameter ranging from 35 to 45 nm, and immunogold EM analysis confirmed that these structures indeed contained HCV core protein (Fig 6, WT, right panel). Similar capsid-containing particles were also detected in 1.13-g/mL fraction from gradient loaded with lysate of cells transfected with the mutated Con1/C3/Sp1mt replicon (Fig 6, compare Sp1mt with WT). By contrast, a lesser amount of material resembling particles was observed in the case of ΔE1E2-mutated replicon, their size ranging from 60 to 150 nm (Fig 6, ΔE1E2, left panel), and immunogold labeling after delipidation showed large and amorphous complexes of core protein presumably representing aggregates (Fig 6, ΔE1E2, right panel). We conclude that inhibition of SP-catalyzed cleavage at the core-E1 junction did not prevent the intracellular formation of non-infectious enveloped particles containing capsids.

## Discussion

In a previous study, we confirmed the sequential processing of HCV core protein first by SP at the core-E1 junction and second by SPP to liberate the mature core protein, and we showed that the SP-catalyzed cleavage is required for the HCV infectious cycle [5]. In the present study, we tried to understand precisely how the processing of core protein by SP is related to HCV infectious cycle. Our results show that SP-catalyzed cleavage at the core-E1 junction is required for the formation of infectious particles and for the release of any HCV particles irrespective of their infectivity. Unexpectedly, however, inhibition of this cleavage did not prevent budding at the ER membrane and intracellular formation of capsid-containing particles; instead, it tended to facilitate the budding process.

Since the development of the HCV infection system, only two publications have reported EM pictures of putative intracellular particles visualized *in situ* in Huh-7 cells. In the first study, the HCV genome had been modified by replacing a part of the hypervariable region 1 of E2 by a tag [11]. It is possible that this mutation disturbs the virus morphogenesis process, allowing EM visualization of particles inside the cells. In the second study, “particles resembling HCV” were observed in endosomal compartments of cells infected with a non-modified virus [34]. It may be proposed that HCV budding and secretion happen so rapidly that neither HCV budding events nor fully formed HCV particles can be routinely visualized within the producing cells at steady state. This is supported by our finding that the amount of infectious particles within the cell was only 0.6% of the amount of extracellular infectious particles; comparable results were reported for other JFH1-derived viruses [44]. Moreover, viral production from infected Huh-7 cells was roughly estimated in the range of one infectious HCV particle per cell per infectious cycle [33], which is in accordance with our data obtained at the peak of infection (Fig 1D: approximately 4 x 10^5^ ffu/mL at day 10 post-transfection with 1 mL of supernatant for 10^6^ cells). Accordingly, imaging such a rare event remains challenging. By contrast, BHK-21 cells transfected with a SFV-based replicon is a choice model to study the early stages of HCV budding: indeed, the budding process is abortive in these cells, which may explain why budding particles accumulate within the cells and can be visualized at steady state by EM [35]. Using this system, we showed that inhibition of SP-catalyzed cleavage at the core-E1 junction did not prevent initiation of HCV budding, but instead tended to promote the budding process at least quantitatively (greater density of viral buds). Qualitatively, most particles also seemed to have moved forwards up to a later stage of budding, although we cannot completely rule out that different protein species used as structural bricks for building the viral particle might influence the shape of the bud. At first glance this was in fact surprising because, when SP-catalyzed cleavage at the core-E1 junction is inhibited, core protein is not allowed to be processed by SPP into its mature form and E1 most certainly remains in its unfolded conformation [5, 45]. Nevertheless, we previously showed that inhibition of SPP-catalyzed cleavage of core protein also tended to promote the budding process [36]. A feature shared by p23 and uncleaved core-E1 precursor but not by p21 is the transmembrane domain at the C-terminus of core, which anchors the protein in the ER lipid bilayer [4], suggesting that it plays a role in the initiation of HCV budding. Indeed, although this domain was reported to be dispensable for infectious virus production when core protein is provided in trans [46], it may be hypothesized that, in the normal, most efficient HCV life cycle where core protein is provided in cis, this domain is important to accumulate and concentrate HCV core protein at the virus budding site in the ER, where HCV envelope glycoproteins do reside owing to ER retention signals, to guarantee the efficient incorporation of all the virus structural proteins into the budding particle [17, 45]. Moreover, the existence of nucleocapsid-free subviral LVPs both *in vitro* produced by cells stably expressing HCV envelope glycoproteins and *in vivo* in the circulating blood of HCV-infected patients suggests that HCV envelope glycoproteins confer at least part of the driving force of budding [17, 47, 48]. Accordingly, the close proximity of HCV core protein and envelope glycoproteins, best achieved in case of a not yet cleaved core-E1 precursor, should be beneficial to core envelopment into budding particles. The requirement of HCV core protein localization at the ER membrane for efficient virus production was recently put forward in view of the predominant localization of the core protein of high-titer viruses, which co-localizes with the viral envelope glycoproteins at the ER, in contrast to the core protein of lower-titer viruses, which accumulates on the surface of LD [44, 49]. The core protein was also found to accumulate on the LD surface in the case of HCV mutants deleted of the envelope glycoproteins or bearing changes in the viral protein p7 or in both p7 and NS2 that diminish core-ER localization, resulting in a defect in intracellular formation of viral particles [42, 49, 50]. Finally, mutation of the palmitoylation site of HCV core protein, which reduced its association with ER membranes, also reduced viral production [51]. Thus, the magnitude of core-ER localization positively correlates with the efficiency of HCV morphogenesis, which may help explain why the presence of core protein in the form of a precursor tends to promote the budding process. In the context of WT virus, however, SP- and SPP-catalyzed cleavages occur so rapidly that at steady state p23 and core-E1 precursor proteins could be detected by western blotting analysis only when using highly sensitive detection systems and very long exposure of the blots (data not shown). Indeed, this is in accordance with the idea of a rare and very rapid budding process.

Whereas core-E1 precursor and p23 are anchored in the ER lipid bilayer by the transmembrane domain at the C-terminus of core, p21, which is devoid of transmembrane domain, is free for trafficking out of the ER and preferentially relocalizes to the surface of LDs [4, 5, 10]. HCV core protein also induces LD rearrangement from a scattered distribution throughout the cytoplasm to a pool tightly linked to ER-derived membranes in the perinuclear region [11, 52]. Numerous studies have reported the importance of core protein-LD interaction in HCV infectious cycle [7, 8, 15]. Indeed, when translocation of HCV core protein to LDs was prevented by different approaches, production of infectious HCV was affected [11–13]. As HCV infectivity is positively correlated with the triglyceride content of viral particles [53, 54], a possible role of core protein-LD interaction may be to regulate the cellular metabolism of triglycerides and perhaps to bring the primary source of triglycerides for incorporation into VLDLs close to the virus assembly site [17]. Furthermore, core protein was shown to recruit the HCV replication complex to LD-associated membranes [11], and an increasing body of evidence has suggested that core protein acts in concert with the non-structural protein NS5A at the surface of LDs to control the switch between viral replication and initiation of viral assembly [7–9]. In heterologous expression systems such as the BHK-21 model, there is no HCV genome replication and thus no need for such a tight regulation, which may explain why viral assembly can be initiated in absence of determinants required in the infectious system in Huh-7 cells. Nevertheless, our work shows that SP-catalyzed cleavage at the core-E1 junction and hence relocation of core protein to LDs are dispensable not only for the initiation of budding in BHK-21 cells, but also for the intracellular formation of capsid-containing particles in Huh-7 cells harboring a full-length HCV replicon. These data do not argue against a specific role of LDs in the production of infectious virus, but indicate that HCV core protein localization to LDs is not a prerequisite for the viral budding step. We thus propose the existence of two pools of HCV core protein: one composed exclusively of p21 and localized at the surface of LD, which may control the switch between viral replication and morphogenesis and/or regulate the intracellular availability of triglycerides, the other constituted of the various forms of core protein (core-E1 precursor, p23, and p21) and localized on the cytosolic side of the ER, which may initiate the viral particle morphogenesis. This would fit with a model in which HCV assembly occurs at ER membranes that may be close to LDs rather than on the LD surface *per se* [14, 16].

Because the most generally admitted model for HCV morphogenesis involves two distinct subcellular localizations for nucleocapsid assembly and budding (LD and ER, respectively), it also implies that these steps necessarily occur sequentially instead of simultaneously; yet this temporal relationship remains uncertain owing to difficulties in imaging such events in HCV-infected cells [15, 16]. Before the development of the infectious system in Huh-7 cell sublines, heterologous expression systems were extensively used to study HCV morphogenesis. Studies *in vitro* showed that assembly of HCV core protein into capsid-like structures can occur in the absence of membranes, requiring only the N-terminal region of the core sequence [55–57]. However, high-ordered multimeric core structures pre-assembled *in vitro* or in cells overexpressing HCV core protein bound to but were not enveloped by membranes *in vitro*, suggesting that nucleocapsid envelopment cannot occur once the assembly step is completed [28]. In yeast overexpressing HCV core protein, cleavage by SPP was not required for production of enveloped nucleocapsid-like particles and could occur after particle envelopment; in fact, the rapidity of SPP-catalyzed cleavage of core protein was positively correlated to production of non-enveloped nucleocapsid-like particles, suggesting that delaying SPP-catalyzed cleavage promotes the viral budding process and also that nucleocapsids cannot be enveloped after completion of assembly [58]. Using heterologous systems for overexpression of HCV structural proteins, we showed in a previous work that inhibition of SPP-catalyzed cleavage of core protein not only did not prevent but instead tended to facilitate the visualization of budding events in mammalian BHK-21 cells and the recovery of HCV-like particles containing capsids from insect cells [36]. In the present work, we not only showed that inhibition of SP-catalyzed cleavage at the core-E1 junction promoted the budding process in BHK-21 cells, but we were also able to recover capsid-containing particles from lysates of Huh-7 cells transfected with a full-length JFH1-derived HCV replicon bearing mutations inhibiting this cleavage. They looked similar to those visualized with the WT counterpart of HCV replicon, which is infectious: indeed, in both cases they appeared as enveloped particles of 60 to 80 nm in diameter containing a capsid of 35 to 45 nm in diameter. This description fits with a report from Gastaminza *et al*., in which isopycnic and velocity ultracentrifugation was used to determine the density (1.15 g/mL) and the size (70 nm in diameter) of intracellular JFH1 particles, respectively [25]. To our knowledge, this is the first report of EM visualization of capsids contained in particles recovered from lysates of Huh-7 cells harboring an infectious HCV replicon. Taken together, the data obtained upon inhibition of core protein processing indicate that assembly and budding can occur simultaneously at ER membranes, and further suggest that this scenario may also happen when core processing is allowed to occur. The mere presence of only a few molecules of p23 or core-E1 precursors not yet processed would not prevent the different forms of core protein (which share the N-terminal region required for oligomerization) from assembling into capsids and would facilitate the simultaneous budding of the assembling capsids. Conversely, the membrane scaffold provided by the ER during the budding process is expected to facilitate the completion of assembly of fully closed capsids [42].

Although inhibition of SP-catalyzed cleavage at the core-E1 junction did not prevent intracellular formation of capsid-containing particles in Huh-7 cells, these were not infectious. Several non-mutually exclusive explanations may account for this observation, as several determinants are required for a nascent viral particle to acquire its infectivity. In particular, a unique feature to be considered for HCV is that infectivity is mainly supported by viral particles of exceptionally low density due to association with triglyceride-rich lipoproteins resembling VLDLs [21, 22, 53, 54, 59]. These LVPs are believed to assemble within the hepatocyte, which is both the primary replication site of HCV and the cell type specialized in the secretion of VLDLs. Although hepatocarcinoma-derived Huh-7 cell lines do not fully reproduce the biogenesis of authentic VLDLs [41], HCV particles produced by these cells were found to be associated with VLDL components [24, 60–62]. Similar to VLDLs, viral particle precursors would undergo lipidation during their intracellular maturation in post-ER compartments before being released into the culture supernatant [18, 23–25]. The mutant particles formed in cells transfected with Con1/C3/Sp1mt replicon are probably not allowed to traffic to post-ER compartments to undergo this lipidation required for gaining infectivity. However, as one of the unresolved issues in HCV morphogenesis is whether the VLDL machinery is involved as early as in the budding step [17], it would be of interest to determine whether association with VLDL components nevertheless occurs in the ER in the case of the budding-competent Sp1mt mutant.

Although we were able to recover capsid-containing particles from lysates of Huh-7.5.1 cells transfected with the full-length Con1/C3/Sp1mt replicon, no viral particles were found in the corresponding cell culture supernatants. We would have expected that, in the absence of secretion, capsid-containing particles would accumulate within the cells. However, no accumulation of core-E1 precursor protein was seen (Fig 1), and despite numerous attempts, observation by EM of Huh-7.5.1 cells transfected with the Con1/C3/Sp1mt replicon did not lead to any visualization of either budding events or viral particles stored within the cells (data not shown). Taken together these data suggest that the capsid-containing particles formed by core-E1 precursor proteins are not allowed to accumulate, probably due to their degradation by the cellular machinery. A recent study showed that when SPP-catalyzed cleavage of core protein is not allowed to occur, p23 is rapidly degraded by the ubiquitin-proteasome degradation pathway through its interaction with the E3 ubiqutin ligase TRC8 [29]. However, such a mechanism is unlikely to apply to core-E1 precursor proteins already incorporated within capsid-containing particles. Unlike the core moiety of core-E1 precursor, the E1 moiety is probably exposed at the surface of particles, consistent with its sensitivity to proteinase K digestion, and it could be recognized as being misfolded by the cellular quality control. Indeed, with their abundant *N*-glycans and disulfide bonds, HCV envelope glycoproteins are subjected to the ER quality control machinery that assists their complex folding, retains misfolded proteins in the ER, and eventually targets them for degradation [17, 45]. In particular, HCV infection was shown to activate the ER-associated degradation pathway that in turn enhances the degradation of the virus envelope glycoproteins, hence limiting the production of viral particles [19]. However, it is difficult to imagine how glycoproteins could be retrotranslocated into the cytosol once they are incorporated into viral particles. Thus, our Sp1mt mutant could be used as a tool in future studies to gain insight into the quality control of newly made HCV particles before they are allowed to progress towards maturation and secretion.

## Supporting information

S1 Appendix Supporting material and methods. (DOCX)

S1 Fig Impact of inhibition of SP-catalyzed cleavage at the core-E1 junction on HCV infectious cycle. Huh-7.5.1 cells were transfected with the full-length HCV RNAs Con1/C3 (WT), Con1/C3/Sp1mt (Sp1mt), or Con1/C3/ΔE1E2 (ΔE1E2). Culture supernatants were harvested and cells were lysed at the indicated days after transfection. (A) Cell lysates were subjected to western blot analysis with antibodies directed against HCV E2 envelope glycoprotein (anti-E2), HCV NS3 protein (anti-NS3) or calnexin (anti-calnexin). Positions on blots of protein molecular mass standards are indicated (in kDa). The same membrane reprobed with the different antibodies is shown. (B) Total RNAs extracted from cell lysates were probed for negative-strand HCV RNA. The threshold of detection of this assay is evaluated at 1.3 log copies per mg of total RNA. (C) Culture supernatants were probed for LDH activity. The threshold of detection of this assay is evaluated at 0.3 log arbitrary units (AU) per mg of total protein. The mean values and standard errors of at least 3 independent experiments are shown.
(PPTX)

S2 Fig Effects of the mutations on HCV polyprotein processing in BHK-21 cells. BHK-21 cells were electroporated with the recombinant RNAs SFV-*lac*Z (LacZ), SFV-*HCV*1b (WT), SFV-*HCV*1b/Sp1mt (Sp1mt), or SFV-*HCV*1b/Sp2mt (Sp2mt). Transfected cells were cultured in the presence or absence of the signal-peptide peptidase (SPP) inhibitor (Z-LL)_2_-ketone at the concentration of 20 mM [(Z-LL)_2_]. Cell lysates were subjected to western blot analysis with mAb against HCV E2 glycoprotein (anti-E2) or HCV core protein (anti-core). Positions on blots of protein molecular mass standards are indicated (in kDa). The same membrane reprobed with the different antibodies is shown.
(PPTX)

S3 Fig Intracellular distribution of WT and mutant HCV core proteins. BHK-21 cells were electroporated with the recombinant RNAs SFV-*lac*Z (LacZ), SFV-HCV1b (WT), SFV-*HCV*1b/Sp1mt (Sp1mt), or SFV-*HCV*1b/Sp2mt (Sp2mt). Transfected cells were fixed, permeabilized, and subjected to double-label immunofluorescence staining for confocal microscopy detection of HCV core protein (red) and marker (green), BODIPY 493/503 (lipid) or calnexin (ER).
(PPTX)

S4 Fig In situ EM visualization of control BHK-21 cells. Representative electron micrographs of ultra-thin sections of BHK-21 cells electroporated with the recombinant SFV-*lac*Z RNA (LacZ). Cells were processed for (A) conventional EM, or (B) immunogold labeling with mAbs against HCV core protein (+ anti-core) or HCV E2 envelope glycoprotein (+ anti-E2).
(PPTX)
