# Supplementary material for: Role of cleavage at the core-E1 junction of hepatitis C virus polyprotein in viral morphogenesis
Source: PLoS One. 2017 Apr 24;12(4):e0175810. doi: 10.1371/journal.pone.0175810 (PMC5402940; doi:10.1371/journal.pone.0175810)
Supplement: S1 Appendix — (DOCX) [file pone.0175810.s001.docx]

S1 Appendix

Supporting Material and Methods

Western blotting

In the case of BHK-21 cells, western blotting was performed as previously described [[1](#_ENREF_1)]. Briefly, cell lysates in reducing sample buffer were electrophoresed in SDS‑15% polyacrylamide gels under reducing conditions, in parallel with protein molecular mass standards (MagicMark^TM^, Invitrogen). Primary antibodies were mAb directed against HCV E2 glycoprotein (AP33, kindly provided by A. H. Patel) or HCV core protein (1856, Virostat).

Quantification of negative-strand HCV RNA

Negative-strand HCV RNA levels were quantified in cell lysates of Huh-7.5.1 cells by means of a strand‑specific reverse transcription real-time polymerase chain reaction technique described previously [[2](#_ENREF_2)].

Cytotoxicity assay

Cytotoxicity was assessed by measuring lactate-dehydrogenase (LDH) activity in supernatants using a Cytotox-96 nonradioactive cytotoxicity assay (Promega) as per manufacturer’s instructions. Results in arbitrary units (AU) were normalized to total intracellular protein amount.

Immunofluorescence microscopy analysis of BHK-21 cells

BHK-21 cells transfected with SFV-based replicon RNAs were seeded immediately after electroporation onto 8‑chamber Lab‑Tek^R^ glass slides (Nunc) at 10^4^ cells per chamber, and cultured in growth medium. All steps of immunofluorescence microscopy preparation were extensively described elsewhere [[3](#_ENREF_3)]. Briefly, cells were fixed with 4% paraformaldehyde and permeabilized with 0.1% Triton X‑100. Primary antibodies were mAb directed against HCV core protein (1856, Virostat) or rabbit antiserum directed against calnexin (C4731, Sigma-Aldrich). BODIPY^R^ 493/503 (Invitrogen, Molecular Probes) was used for LD staining.

Supporting References

1. Vauloup-Fellous C, Pène V, Garaud-Aunis J, Harper F, Bardin S, Suire Y, et al. Signal peptide peptidase catalysed cleavage of hepatitis C virus core protein is dispensable for virus budding, but destabilizes the viral capsid. The Journal of biological chemistry. 2006;281(38):27679-92.

2. Carrière M, Pène V, Breiman A, Conti F, Chouzenoux S, Meurs E, et al. A novel, sensitive, and specific RT-PCR technique for quantitation of hepatitis C virus replication. Journal of medical virology. 2007;79(2):155-60. doi: 10.1002/jmv.20773. PubMed PMID: 17177304.

3. Pène V, Hernandez C, Vauloup-Fellous C, Garaud-Aunis J, Rosenberg AR. Sequential processing of hepatitis C virus core protein by host cell signal peptidase and signal peptide peptidase: a reassessment. Journal of viral hepatitis. 2009;16(10):705-15. doi: 10.1111/j.1365-2893.2009.01118.x. PubMed PMID: 19281487.
