# Supplementary material for: Role of cleavage at the core-E1 junction of hepatitis C virus polyprotein in viral morphogenesis
Source: PLoS One. 2017 Apr 24;12(4):e0175810. doi: 10.1371/journal.pone.0175810 (PMC5402940; doi:10.1371/journal.pone.0175810)
Supplement: S1 Fig — Huh-7.5.1 cells were transfected with the full-length HCV RNAs Con1/C3 (WT), Con1/C3/Sp1mt (Sp1mt), or Con1/C3/ΔE1E2 (ΔE1E2). Culture supernatants were harvested and cells were lysed at the indicated days after transfection. (A) Cell lysates were subjected to western blot analysis with antibodies directed against HCV E2 envelope glycoprotein (anti-E2), HCV NS3 protein (anti-NS3) or calnexin (anti-calnexin). Positions on blots of protein molecular mass standards are indicated (in kDa). The same membrane reprobed with the different antibodies is shown. (B) Total RNAs extracted from cell lysates were probed for negative-strand HCV RNA. The threshold of detection of this assay is evaluated at 1.3 log copies per mg of total RNA. (C) Culture supernatants were probed for LDH activity. The threshold of detection of this assay is evaluated at 0.3 log arbitrary units (AU) per mg of total protein. The mean values and standard errors of at least 3 independent experiments are shown. (PPTX) [file pone.0175810.s002.pptx]

## Slide 1
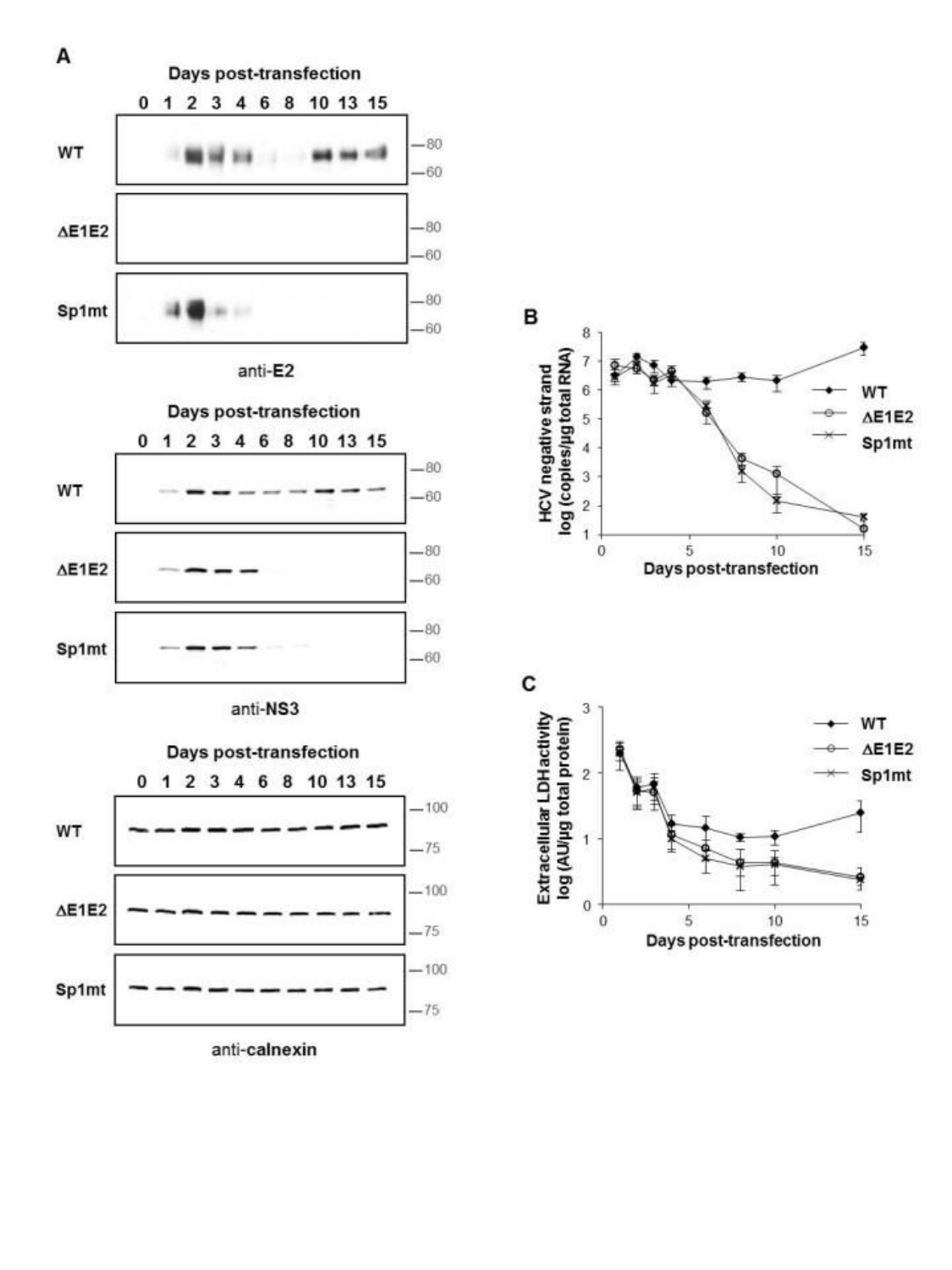

## Slide 2
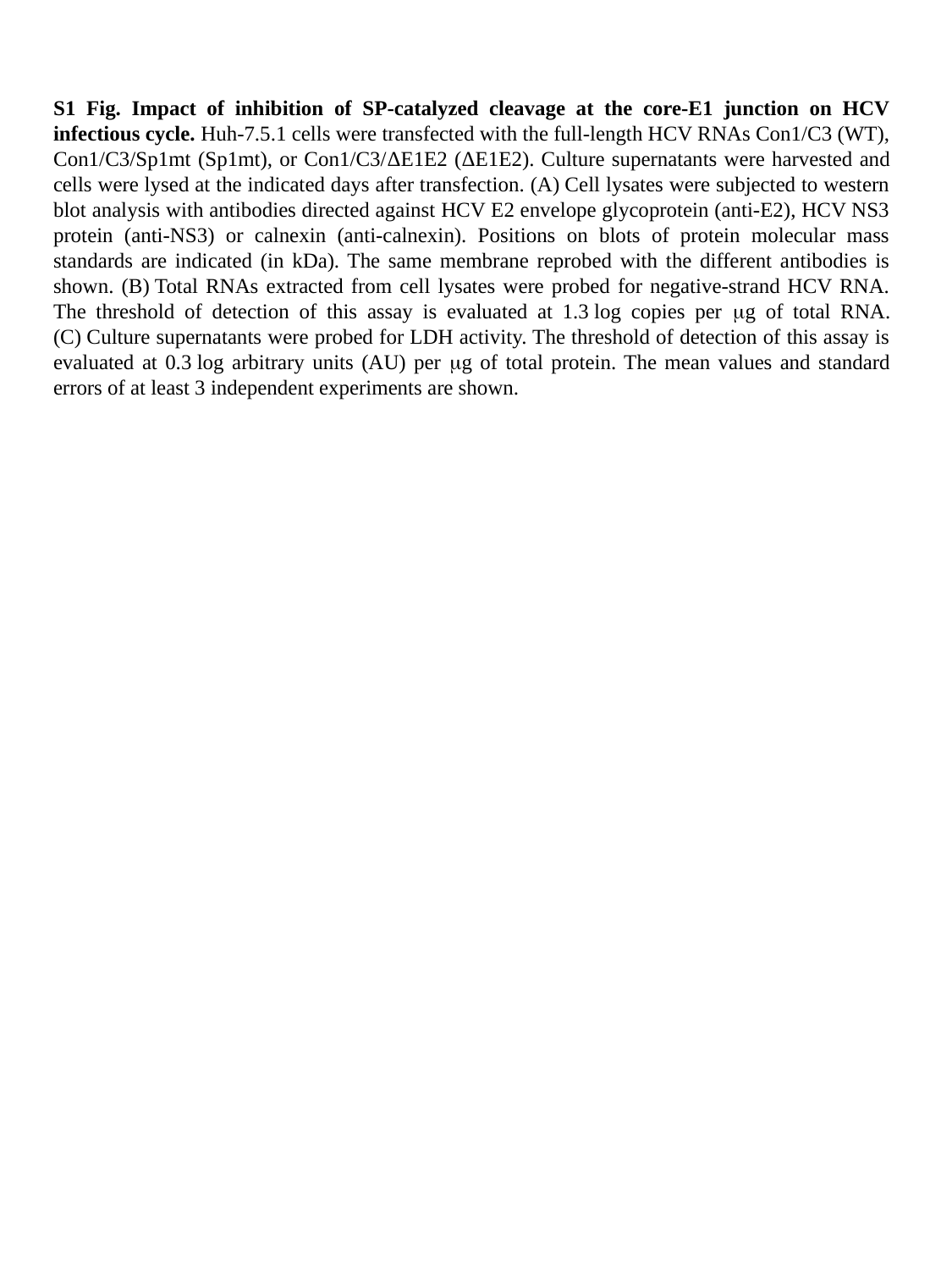

S1 Fig. Impact of inhibition of SP-catalyzed cleavage at the core-E1 junction on HCV infectious cycle. Huh-7.5.1 cells were transfected with the full-length HCV RNAs Con1/C3 (WT), Con1/C3/Sp1mt (Sp1mt), or Con1/C3/ΔE1E2 (ΔE1E2). Culture supernatants were harvested and cells were lysed at the indicated days after transfection. (A) Cell lysates were subjected to western blot analysis with antibodies directed against HCV E2 envelope glycoprotein (anti-E2), HCV NS3 protein (anti-NS3) or calnexin (anti-calnexin). Positions on blots of protein molecular mass standards are indicated (in kDa). The same membrane reprobed with the different antibodies is shown. (B) Total RNAs extracted from cell lysates were probed for negative-strand HCV RNA. The threshold of detection of this assay is evaluated at 1.3 log copies per mg of total RNA. (C) Culture supernatants were probed for LDH activity. The threshold of detection of this assay is evaluated at 0.3 log arbitrary units (AU) per mg of total protein. The mean values and standard errors of at least 3 independent experiments are shown.
