# Supplementary material for: Role of cleavage at the core-E1 junction of hepatitis C virus polyprotein in viral morphogenesis
Source: PLoS One. 2017 Apr 24;12(4):e0175810. doi: 10.1371/journal.pone.0175810 (PMC5402940; doi:10.1371/journal.pone.0175810)
Supplement: S2 Fig — BHK-21 cells were electroporated with the recombinant RNAs SFV-lacZ (LacZ), SFV-HCV1b (WT), SFV-HCV1b/Sp1mt (Sp1mt), or SFV-HCV1b/Sp2mt (Sp2mt). Transfected cells were cultured in the presence or absence of the signal-peptide peptidase (SPP) inhibitor (Z-LL)2-ketone at the concentration of 20 mM [(Z-LL)2]. Cell lysates were subjected to western blot analysis with mAb against HCV E2 glycoprotein (anti-E2) or HCV core protein (anti-core). Positions on blots of protein molecular mass standards are indicated (in kDa). The same membrane reprobed with the different antibodies is shown. (PPTX) [file pone.0175810.s003.pptx]

## Slide 1
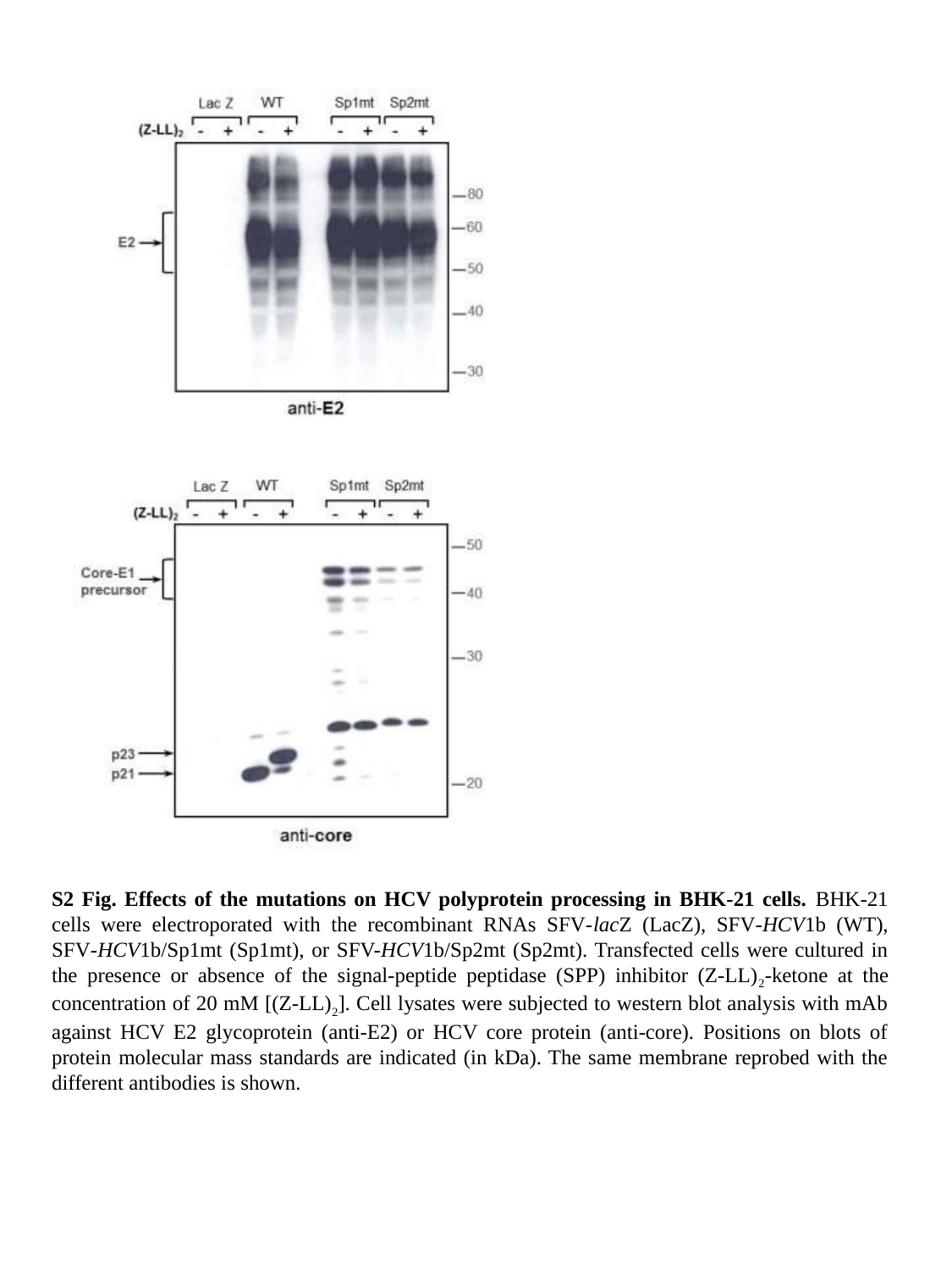

S2 Fig. Effects of the mutations on HCV polyprotein processing in BHK-21 cells. BHK-21 cells were electroporated with the recombinant RNAs SFV‑lacZ (LacZ), SFV‑HCV1b (WT), SFV‑HCV1b/Sp1mt (Sp1mt), or SFV-HCV1b/Sp2mt (Sp2mt). Transfected cells were cultured in the presence or absence of the signal-peptide peptidase (SPP) inhibitor (Z-LL)2-ketone at the concentration of 20 mM [(Z-LL)2]. Cell lysates were subjected to western blot analysis with mAb against HCV E2 glycoprotein (anti-E2) or HCV core protein (anti-core). Positions on blots of protein molecular mass standards are indicated (in kDa). The same membrane reprobed with the different antibodies is shown.
