# Supplementary material for: Role of cleavage at the core-E1 junction of hepatitis C virus polyprotein in viral morphogenesis
Source: PLoS One. 2017 Apr 24;12(4):e0175810. doi: 10.1371/journal.pone.0175810 (PMC5402940; doi:10.1371/journal.pone.0175810)
Supplement: S3 Fig — BHK-21 cells were electroporated with the recombinant RNAs SFV-lacZ (LacZ), SFV-HCV1b (WT), SFV-HCV1b/Sp1mt (Sp1mt), or SFV-HCV1b/Sp2mt (Sp2mt). Transfected cells were fixed, permeabilized, and subjected to double-label immunofluorescence staining for confocal microscopy detection of HCV core protein (red) and marker (green), BODIPY 493/503 (lipid) or calnexin (ER). (PPTX) [file pone.0175810.s004.pptx]

## Slide 1
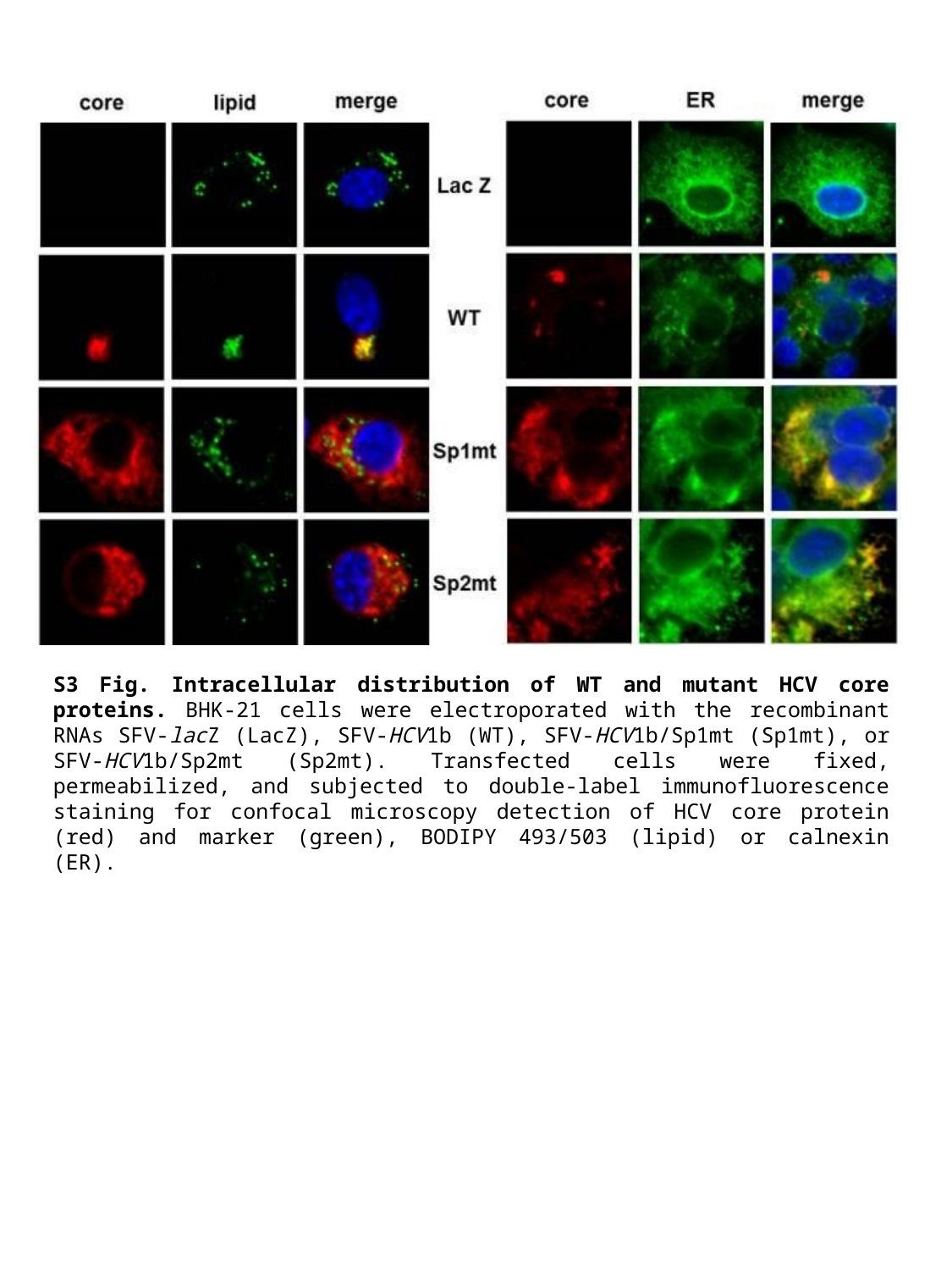

S3 Fig. Intracellular distribution of WT and mutant HCV core proteins. BHK-21 cells were electroporated with the recombinant RNAs SFV‑lacZ (LacZ), SFV‑HCV1b (WT), SFV‑HCV1b/Sp1mt (Sp1mt), or SFV-HCV1b/Sp2mt (Sp2mt). Transfected cells were fixed, permeabilized, and subjected to double-label immunofluorescence staining for confocal microscopy detection of HCV core protein (red) and marker (green), BODIPY 493/503 (lipid) or calnexin (ER).
