# Supplementary material for: Role of cleavage at the core-E1 junction of hepatitis C virus polyprotein in viral morphogenesis
Source: PLoS One. 2017 Apr 24;12(4):e0175810. doi: 10.1371/journal.pone.0175810 (PMC5402940; doi:10.1371/journal.pone.0175810)
Supplement: S4 Fig — Representative electron micrographs of ultra-thin sections of BHK-21 cells electroporated with the recombinant SFV-lacZ RNA (LacZ). Cells were processed for (A) conventional EM, or (B) immunogold labeling with mAbs against HCV core protein (+ anti-core) or HCV E2 envelope glycoprotein (+ anti-E2). (PPTX) [file pone.0175810.s005.pptx]

## Slide 1
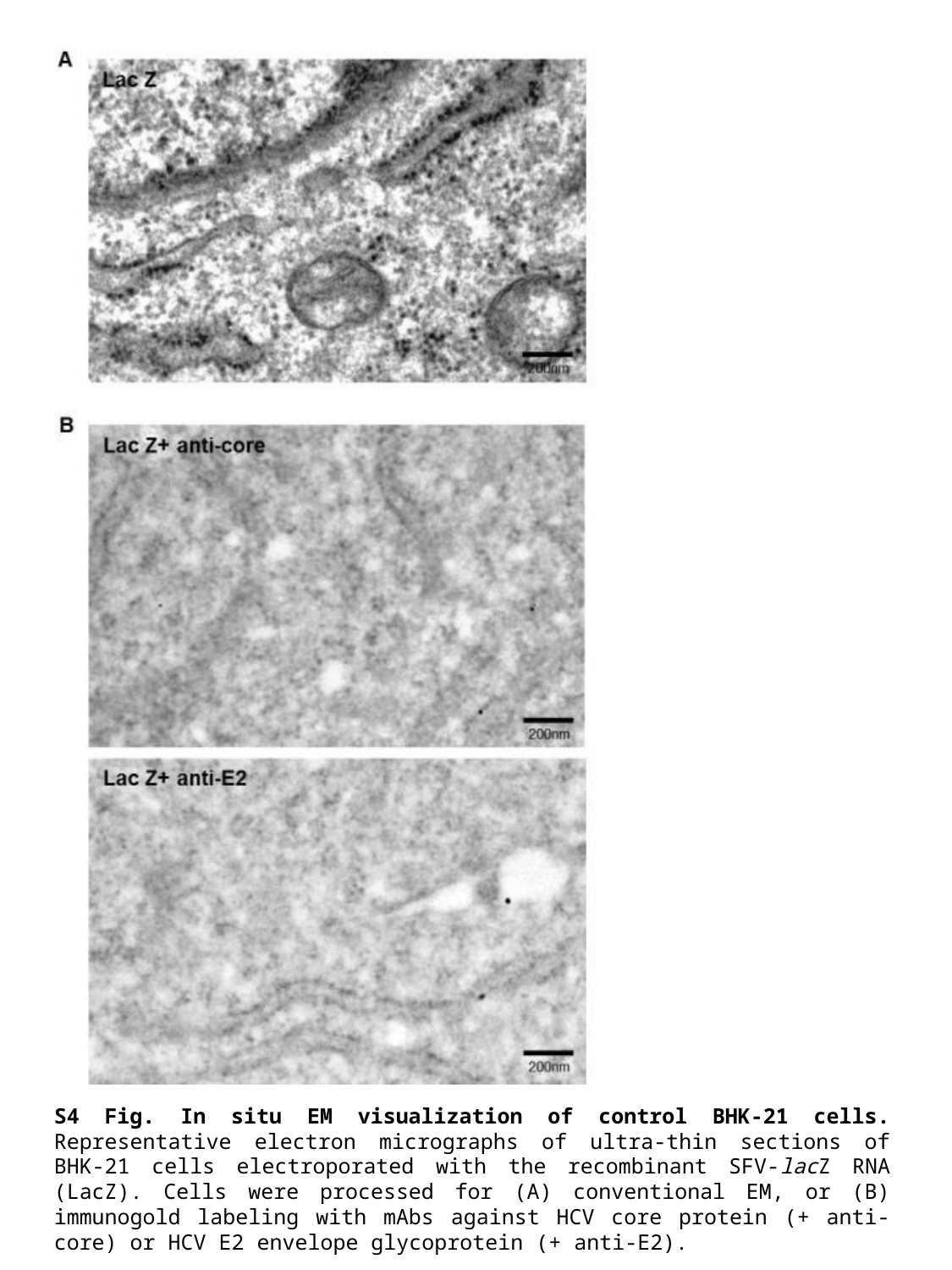

S4 Fig. In situ EM visualization of control BHK-21 cells. Representative electron micrographs of ultra-thin sections of BHK-21 cells electroporated with the recombinant SFV‑lacZ RNA (LacZ). Cells were processed for (A) conventional EM, or (B) immunogold labeling with mAbs against HCV core protein (+ anti-core) or HCV E2 envelope glycoprotein (+ anti-E2).
